# Surgical Pleth Index as a Potentially Useful and Noninvasive Tool for Assessing Tracheal Intubation Conditions in Female Patients During Neuromuscular Blockade‐Free Anesthesia

**DOI:** 10.1155/emmi/7863986

**Published:** 2026-03-09

**Authors:** Jiale Chen, Zhihao Pan, Jinwei Zheng

**Affiliations:** ^1^ Department of Anesthesiology, The Affiliated Lihuili Hospital of Ningbo University, Ningbo, 315046, Zhejiang, China; ^2^ Department of Anesthesiology, Ningbo No. 2 Hospital, Ningbo, 315010, Zhejiang, China, nbws.gov.cn

**Keywords:** neuromuscular blocking agents, predictive value, surgical pleth index, tracheal intubation

## Abstract

**Background:**

Neuromuscular blocking agents (NMBAs) are commonly used during tracheal intubation to ensure smoother procedural conditions, but they are associated with the risk of prolonged paralysis and respiratory complications. This study explores the relationship between the surgical pleth index (SPI) measured immediately before intubation and tracheal intubation conditions in patients who did not receive neuromuscular blockers, along with the predictive value of the SPI in these conditions.

**Methods:**

A total of 100 surgical patients (excluded 17 patients who did not meet the eligibility criteria) undergoing elective surgery under general anesthesia at The Affiliated Lihuili Hospital of Ningbo University between November 2021 and June 2022 were included, and key parameters, including systolic blood pressure (SBP), diastolic blood pressure (DBP), heart rate (HR), and SPI, were measured at different time points (T0 to T5).

**Results:**

At T2, significant reductions in SBP, DBP, HR, and SPI were observed compared to baseline (T0, after the completion of 6 mL/kg of lactate Ringer’s solution infusion) and preintubation values (T1, at the time of loss of consciousness) (*p* < 0.05). Postintubation, both SBP and DBP significantly increased at T5 (3 min after intubation) compared to T2 (immediately before intubation) (*p* < 0.05). The patients were classified into “excellent” and “good” groups based on their intubation conditions. SPI values at T3 (immediately after intubation), T4 (1 min after intubation), and T5 were significantly higher in the “good” group compared to the “excellent” group (*p* < 0.05). Post hoc sex‐stratified receiver operating characteristic (ROC) analysis showed an area under the curve (AUC) of 0.713 (*p* = 0.037) and 95% confidence interval (CI) (0.539–0.887), indicating moderate predictive value for the SPI in assessing tracheal intubation conditions.

**Conclusions:**

The SPI proves to be a potentially useful and noninvasive tool for evaluating tracheal intubation conditions in female patients without the use of NMBAs.

## 1. Introduction

Intubation conditions are defined as the overall state of a patient’s preparedness for tracheal intubation, which is evaluated based on several indicators such as depth of anesthesia. Favorable intubation conditions help reduce the risks associated with airway manipulation during surgical procedures. Muscle relaxants are commonly administered during induction of general anesthesia to optimize intubation conditions [1]. Nonetheless, their use carries the potential for various adverse effects, including allergic reactions, malignant hyperthermia, and postoperative myalgia, as well as nausea and vomiting [2–4]. In particular, patients with certain neuromuscular disorders, such as myasthenia gravis, and pediatric populations may experience delayed respiratory recovery due to the residual effects of muscle relaxants, which can impair swallowing function and increase the risk of aspiration and hypoxemia [5]. Additionally, the use of muscle relaxants complicates surgeries that require intraoperative nerve signal monitoring, such as recurrent laryngeal nerve monitoring during thyroidectomy or facial nerve monitoring during acoustic neuroma surgery, and these drugs can interfere with nerve signal transmission, leading to monitoring failures [6, 7], which may result in incorrect intraoperative decisions and negatively affect surgical outcomes [8]. Omitting neuromuscular blocking agents (NMBAs) offers the potential advantage of avoiding serious complications such as residual neuromuscular blockade and postoperative pulmonary compromise. This approach is particularly suitable for short surgical procedures or in patients with an elevated risk of perioperative respiratory adverse events.

The Enhanced Recovery After Surgery (ERAS) protocols aim to optimize surgical processes and accelerate patient recovery by minimizing perioperative stress [9, 10]. Limiting the use of muscle relaxants during tracheal intubation aligns with ERAS principles [11], as it helps to reduce the adverse effects associated with these drugs, such as postoperative muscle pain and prolonged recovery times [12]. This study thus explores an objective indicator for predicting tracheal intubation conditions without NMBAs, consistent with ERAS goals.

Pain, being inherently subjective, presents a significant challenge in clinical quantification, particularly during general anesthesia, where direct measurement of analgesic efficacy remains elusive [13]. Traditionally, analgesia has been assessed based on nonspecific autonomic responses, including changes in blood pressure, heart rate (HR), sweating, and lacrimation [14]. However, recent advancements have led to the development of more objective tools for pain monitoring in clinical practice, including the analgesia nociception index (ANI), skin conductance (SC), nociception level index (NOL), surgical pleth index (SPI), and pupil diameter measurement [15]. The SPI, introduced in 2007, is a promising tool designed to monitor the balance between surgical pain stimuli and analgesic effects during anesthesia [16] as it provides a quantitative measure of analgesia by analyzing photoplethysmographic pulse wave amplitude (PPGA) and heart beat interval (HBI) from the plethysmographic signal captured by the pulse oxygen saturation (SpO_2_) sensor, with values ranging from 0 to 100 [17]. Among various objective tools for pain monitoring, the SPI was selected for this study due to its unique advantages. Compared to other indices such as ANI and NOL, SPI is derived from PPGA and HBI, which are easily accessible through routine SpO_2_ sensors without additional equipment requirements. Moreover, the SPI has been widely validated in perioperative settings for monitoring the balance between surgical pain stimuli and analgesic effects, showing good feasibility and stability in clinical practice. In contrast, some other indices may have higher technical requirements for measurement or limited data in non‐neuromuscular blockade anesthesia scenarios, making the SPI a more suitable choice for this study.

Given that endotracheal intubation is a significant nociceptive stimulus, the SPI has been utilized to assess the balance between pain stimuli and analgesic administration during general anesthesia. This study aims to investigate an objective indicator capable of predicting tracheal intubation conditions in the absence of NMBAs, with the goal of ensuring optimal intubation conditions. The primary objective is to explore the relationship between SPI values immediately before intubation and tracheal intubation conditions, and to evaluate the predictive utility of the SPI in this context.

## 2. Methods

### 2.1. Participants

The study protocol was approved by the Research Ethics Committee of the Affiliated Lihuili Hospital of Ningbo University (Ethical Application Reference: KY2021SL179‐01, Ningbo, China) on 29 October 2021. All methods were conducted in accordance with relevant ethical guidelines, regulations, and Consolidated Standards of Reporting Trials (CONSORT) recommendations. Prior to participation, written informed consent was obtained from all patients and/or their legal guardians.

**FIGURE 1:**
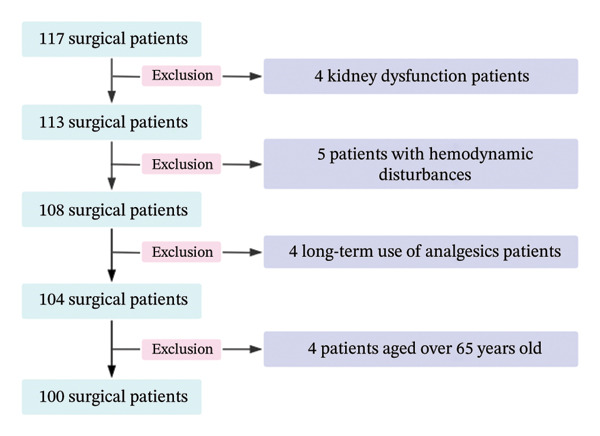
Flowchart illustrating the patient selection and exclusion process for the study.

Initially, 117 patients were considered for inclusion, but after a series of screenings, 100 patients were ultimately enrolled in the study (Figure 1). These participants underwent elective surgery under general anesthesia at The Affiliated Lihuili Hospital of Ningbo University (Ningbo, China) between November 2021 and June 2022. The cohort comprised 47 males and 53 females.

The study inclusion criteria were: (1) age between 18 and 65 years, (2) having an American Society of Anesthesiologists (ASA) physical status classification of 1 or 2, and (3) undergoing elective surgery under general anesthesia. Cases were excluded if they had: (1) severe cardiovascular disease, (2) significant liver or kidney dysfunction, (3) airway hyperresponsiveness, (4) known allergies to study medications, (5) body mass index (BMI) greater than 30 kg/m^2^, (6) risk of esophageal reflux, (7) anticipated intubation difficulties, (8) loose teeth, (9) long‐term preoperative use of β‐blockers, agonists, or clonidine, (10) chronic use of opioid or non‐opioid analgesics, (11) history of peripheral or central nervous system disorders, (12) arrhythmia or pacemaker implantation, (13) baseline mean arterial pressure (MAP) > 120 mmHg or < 60 mmHg, and (14) baseline HR > 100 beats per minute (bpm) or < 45 bpm.

The sample size was determined to ensure sufficient statistical power to detect a clinically significant difference in the primary outcome, which was the SPI during intubation. An a priori power analysis conducted using G∗Power software (version 3.1.9.7, Franz Faul, University Kiel, Kiel, SH, Germany) indicated that 100 patients would provide at least 80% power to detect a medium effect size with a 5% significance level as detailed below.

### 2.2. Hamilton Anxiety Rating Scale (HARS) Psychological Assessment

Before surgery, all 100 patients underwent psychological assessment using the HARS. The scoring system was as follows: 0–7 points indicated no anxiety, 8–17 points indicated mild to moderate anxiety, 18–24 points indicated moderate anxiety, and 25 or more points indicated severe anxiety. The assessment was conducted according to the methodology described by Sulastri et al. [18].

### 2.3. Anesthesia Management

Prior to anesthesia induction, all patients were required to fast for 8 h and abstain from consuming liquids for 2 h. The term “consuming liquids” was defined as the complete avoidance of all liquid substances, including but not limited to water, beverages, soups, juices, dairy products, and alcoholic drinks. In the anesthesia preparation room, a peripheral intravenous line was established 30 min before the operation, and 6 mL/kg of sodium lactate Ringer’s solution was infused.

Upon entry into the operating theater, monitoring was initiated using equipment for electrocardiogram, blood pressure, HR, oxygen saturation (measured in the limb without venous access), SPI, and bispectral index (BIS). Anesthesia induction began with the administration of 2 mg of midazolam. Following this, propofol was slowly infused at a dose of 2 mg/kg. After a 60‐s interval, 4 μg/kg of remifentanil was administered over a 30‐s period. By 90 s, when both propofol and remifentanil reached their peak efficacy, the BIS was maintained between 40 and 60 to facilitate optimal conditions for tracheal intubation [19].

If a patient’s HR decreased below 45 bpm during induction, atropine was administered intravenously (initial dose 0.5 mg, with additional 0.25 mg boluses if needed, maximum total dose 0.75 mg for patients ≥ 60 years). If MAP fell below 60 mmHg, norepinephrine (10–30 μg) was administered. In cases of concurrent hypotension and bradycardia, ephedrine (6 mg) was administered.

### 2.4. Data Collection

The following patient demographics were recorded: general physical condition, sex, age, and weight. Additionally, the systolic blood pressure (SBP), diastolic blood pressure (DBP), HR, and SPI values were measured at several time points during the perioperative period: (1) after the completion of 6 mL/kg of lactate Ringer’s solution infusion (T0), (2) at the time of loss of consciousness (T1), (3) immediately before intubation (T2), (4) immediately after intubation (T3), (5) 1 min after intubation (T4), and (6) 3 min after intubation (T5). T2 measurements were completed before any intubation attempt, as rigorously enforced by the protocol. T2 was explicitly recorded after achieving target BIS (40–60) but before laryngoscope contact with the oropharynx, with a median interval of 12 s (IQR: 8–15 s) between T2 completion and laryngoscopy initiation.

Endotracheal intubation was assisted with a complementary metal oxide semiconductor (CMOS) video laryngoscope. All intubations were performed by experienced anesthesiologists with a minimum of 5 years of practice and over 100 cases of airway management. The dosage and timing of remifentanil administration during induction, along with the assessment of tracheal intubation conditions, were evaluated using the validated scoring system developed by Erhan et al. [20] (Table 1). This system quantifies intubation quality based on seven criteria: ease of mask ventilation, jaw relaxation, difficulty of laryngoscopy, vocal cord position, intensity of coughing, limb movement, and response to cuff inflation. Each parameter is scored from 1 (optimal) to 4 (suboptimal). Overall intubation conditions were classified as “excellent” if all criteria scored 1, “good” if mask ventilation scored 1 and all others were ≤ 2, and “poor” if any criterion scored ≥ 3. The use of this standardized instrument ensures objective and reproducible assessment of intubation conditions. In cases where intubation was unsuccessful, intravenous rocuronium bromide (0.6 mg/kg) was administered, calculated based on the patient’s actual body weight, to facilitate successful intubation. Any adverse effects associated with this intervention were also recorded.

**TABLE 1:** Intubating condition score.

Variables	Score			
	1	2	3	4
Mask ventilation (A)	Easy	Difficult	Impossible	—
Jaw relaxation (B)	Complete	Slight tone	Stiff	Rigid
Laryngoscopy (C)	Easy	Fair	Difficult	Impossible
Vocal cord position (D)	Open	Moving	Closing	Closed
Coughing (E)	None	Slight	Moderate	Severe
Limb movement (F)	None	Slight	Moderate	Severe
Cuff response (G)	None	Slight	Moderate	Severe

### 2.5. Measurement of SPI

The SPI is a noninvasive, dimensionless score that ranges from 0 to 100 and is used to estimate intraoperative nociception. It is calculated based on the photoplethysmographic (PPG) analysis of pulse wave amplitude and HBI. The SPI is determined using the following equation: SPI = 100 − (0.33 × HBI + 0.67 × PPGA), where PPGA refers to the amplitude of the PPG waveform and HBI represents the time interval between heartbeats. Higher SPI values indicate a greater nociceptive (stress) response, while lower values reflect reduced nociceptive activity. In this study, a pulse oximeter was attached to the patient’s finger to record PPG signals, and SPI values were monitored at the designated time points (T0 to T5).

### 2.6. Monitoring Respiratory Rate (RR), End‐Tidal CO_2_ (EtCO_2_), and Oxygen Saturation (SpO_2_) During Surgery

Continuous monitoring of key respiratory parameters, including RR, EtCO_2_, and SpO_2_, is essential for ensuring patient safety and optimal anesthesia management during surgery. RR was monitored using an RR monitor, which counts the number of breaths per minute, EtCO_2_ was measured through capnography, which provides a graphical representation of CO_2_ levels throughout the breathing cycle, reflecting the patient’s ventilatory status, and SpO_2_ was assessed noninvasively using a pulse oximeter, typically placed on the finger or earlobe, to determine the percentage of hemoglobin saturated with oxygen, which allowed for adjusting ventilation settings, assessing respiratory efficiency, and detecting potential complications early in the surgical process.

### 2.7. Cortisol and C‐Reactive Protein (CRP)

Blood samples were collected at time points T1 and T5 using non‐anticoagulant tubes to obtain whole blood. The samples were allowed to clot at room temperature for 30 min. After clotting, the samples were centrifuged at 3000–3500 rpm for 15 min at 4°C to separate the serum. The concentrations of cortisol (Beyotime, PC100) and CRP (Beyotime, PC100) in the serum were measured using enzyme‐linked immunosorbent assay (ELISA) kits.

### 2.8. Statistical Analysis

The sample size was determined based on the following justified assumptions. First, a medium effect size (Cohen’s *d* = 0.5) was assumed, which is consistent with previous studies investigating the predictive value of physiological indices for intubation conditions. Second, the group ratio of “excellent” to “good” intubation conditions was set at 3:1, based on preliminary clinical observations in our institution showing that approximately 77.5% of patients without NMBAs achieve “excellent” intubation conditions. Third, a one‐tailed test was used because our hypothesis was directional: we predicted that lower SPI values would be associated with better intubation conditions, which is supported by the theoretical basis of SPI reflecting nociceptive responses. An a priori power analysis using PASS11 software (Power Analysis and Sample Size Software, Version 11.0.8; NCSS, LLC, Kaysville, UT, USA) indicated that 100 patients would provide at least 80% power to detect the assumed medium effect size with a 5% significance level, justifying the final sample size.

Statistical analyses were performed using IBM SPSS Statistics 26.0 (Chicago, IL, USA). Continuous data were expressed as mean ± standard deviation (SD). Comparisons of blood pressure, HR, and SPI at different time points were made using one‐way analysis of variance (ANOVA). To compare SPI values between the “excellent” and “good” subgroups at different times, a general linear model with repeated measures was employed. Given the potential gender differences in physiological responses to anesthesia and nociception, a post hoc sex‐stratified receiver operating characteristic (ROC) analysis was conducted as an exploratory analysis to investigate whether the predictive value of SPI varies by gender. This analysis was not prespecified in the study protocol but was initiated based on preliminary observations of gender‐related differences in SPI values during data analysis. The calibration ability of the predictive model was evaluated using the Hosmer–Lemeshow goodness‐of‐fit test.

## 3. Results

### 3.1. Participant Demographic Information

All the 100 eligible patients completed the study, and their general clinical statistics are summarized in Table 2. Data analysis indicated that the mean age of the participants was 48 years, with 47 males and 53 females, and the average weight was 64.4 kg. According to the ASA physical status classification, 71% of the patients were classified as ASA I, while 29% were classified as ASA II.

**TABLE 2:** Demographic data of participants.

Patient characteristics	Values ($\overset{¯}{x}\pm S$ or %)
Age (yr)	48.0 ± 11.7
Gender (%)	
Male	47
Female	53
ASA classification (%)	
I	71
II	29
Weight (kg)	64.4 ± 12.3

### 3.2. HARS Psychological Assessment

**FIGURE 2:**
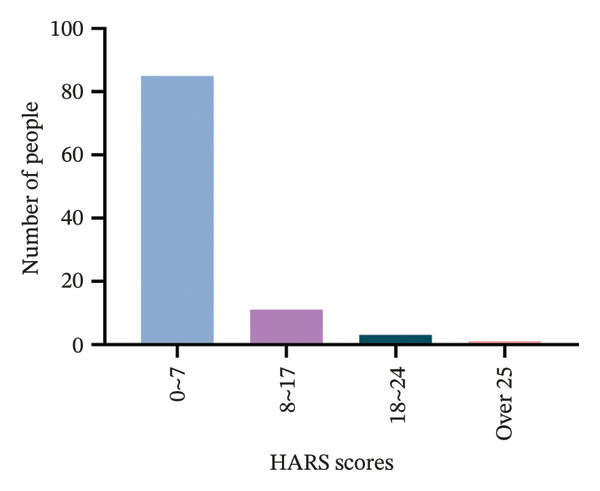
The Hamilton anxiety rating scale (HARS) for categorizing the anxiety of the participants into four levels.

Preoperative anxiety levels were assessed using the HARS (Figure 2). Among the 100 patients, 85 exhibited no anxiety, 11 had mild anxiety, 3 had moderate anxiety, and 1 had severe anxiety.

### 3.3. Changes in SBP, DBP, HR, and SPI

Significant changes in vital signs were observed at various time points (Figure 3). We found that SBP, DBP, HR, and all significantly decreased at T2 compared to T0 and T1 (*p* < 0.05). Following tracheal intubation, SBP and DBP significantly increased at T5 compared to T2 (*p* < 0.05). However, no significant differences were observed in HR and SPI between T2 and T5 (*p* > 0.05).

FIGURE 3Trends in hemodynamic data and SPI during the peri‐intubation period. (a) Systolic blood pressure (SBP), (b) diastolic blood pressure (DBP), (c) heart rate (HR), and (d) surgical pleth index (SPI) measured at six time points: T0 (after completion of 6 mL/kg lactate Ringer’s solution infusion), T1 (time of loss of consciousness), T2 (immediately before intubation), T3 (immediately after intubation), T4 (1 min after intubation), and T5 (3 min after intubation). Data are presented as mean ± SD. Significant differences between time points are indicated by different letters (*p* < 0.05); identical letters indicate no significant difference.

**Figure:**
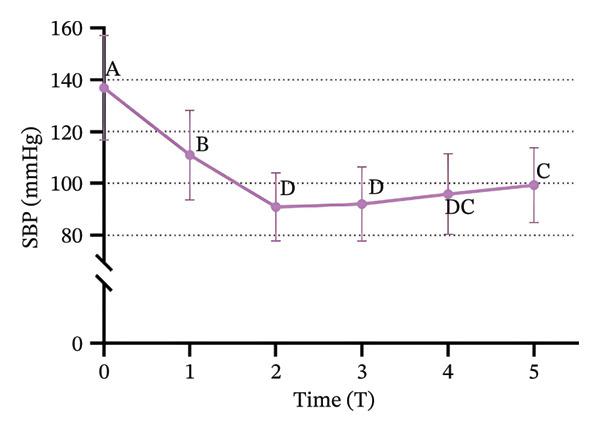
(a)

**Figure:**
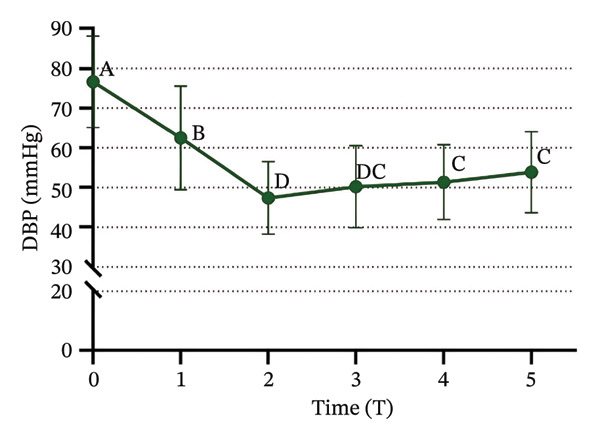
(b)

**Figure:**
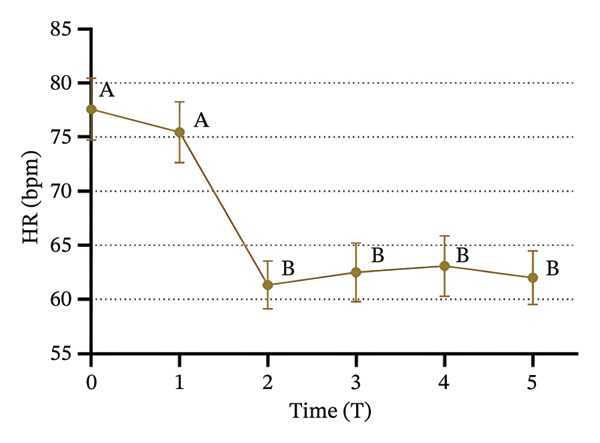
(c)

**Figure:**
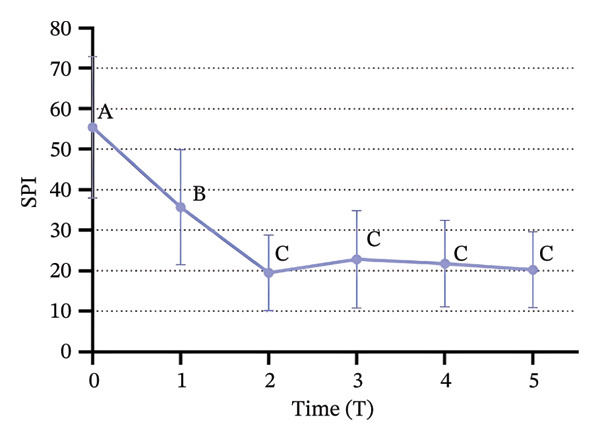
(d)

### 3.4. Comparison of SPI Values Between “Excellent” and “Good” Tracheal Intubation Conditions

**FIGURE 4:**
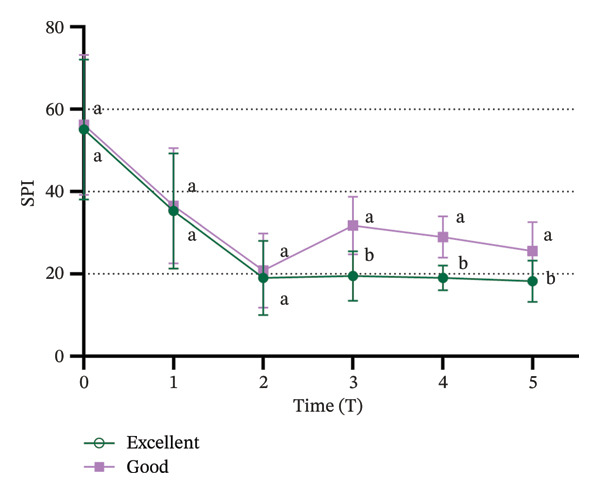
Comparison of SPI between patients with “excellent” and “good” intubation conditions after tracheal intubation. Significant differences in SPI between the “excellent” and “good” groups are indicated by the absence of identical letters at each time point (*p* < 0.05). SPI: surgical pleth index.

All patients successfully underwent endotracheal intubation without any adverse reactions. Of the 100 patients, 58 were rated with “excellent” intubation conditions, while 42 were rated as “good”. No patients were rated as “poor”. A significant difference in SPI values was observed between the “excellent” and “good” intubation condition groups (*p* < 0.01). As shown in Figure 4, SPI values at T3, T4, and T5 were significantly higher in the “good” group compared to the “excellent” group (*p* < 0.05).

### 3.5. Intraoperative Monitoring of RR, EtCO_2_, and SpO_2_ During Surgery

**FIGURE 5:**
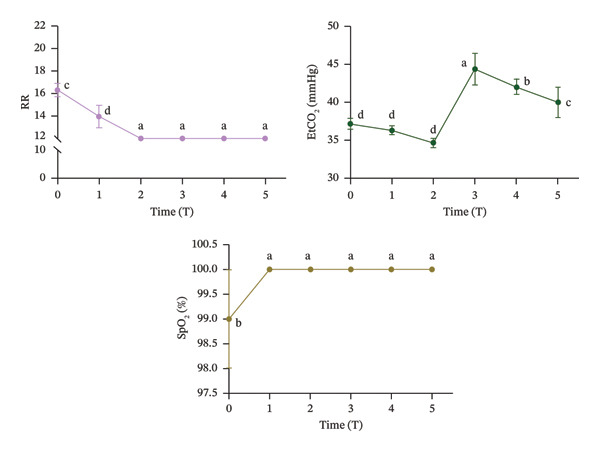
Intraoperative monitoring of RR, EtCO_2_, and SpO_2_ during surgery. Significant differences between time points are indicated by the absence of identical letters (*p* < 0.05).

As illustrated in Figure 5, RR at T2 was significantly lower than at T0 (*p* < 0.05) and subsequently remained stable at 12 bpm (breaths per minute) throughout T2 to T5. No significant difference was found in EtCO_2_ levels between T0 and T2 (*p* > 0.05). However, EtCO_2_ levels significantly increased at T3, exceeding those observed at all other time points (*p* < 0.05). SpO_2_ increased significantly at T1 compared to T0 (*p* < 0.05) and remained unchanged at 100% from T1 to T5 (*p* < 0.05).

### 3.6. Comparison of Cortisol and CRP Levels in Blood

**FIGURE 6:**
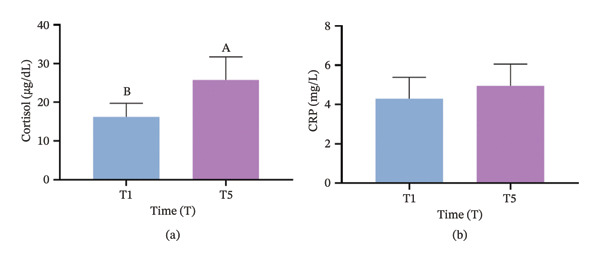
Comparison of hormonal levels during surgery. (a) Cortisol and (b) CRP. Significant differences between time points are indicated by the absence of identical letters (*p* < 0.05).

As illustrated in Figure 6, cortisol levels at T5 were significantly higher compared to T1 (*p* < 0.05). In contrast, the difference in CRP levels between T1 and T5 was not statistically significant (*p* > 0.05).

### 3.7. ROC Analysis

**FIGURE 7:**
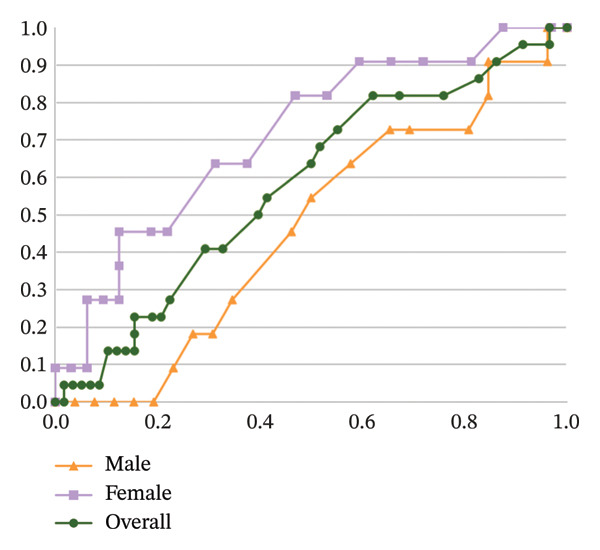
ROC curve analysis of immediate pre‐intubation SPI values for predicting tracheal intubation conditions in different patient populations.

To assess the predictive capacity of the SPI for tracheal intubation conditions, ROC analysis was conducted (Figure 7). For the entire cohort, the SPI did not demonstrate a significant predictive value, yielding an AUC of 0.579 (*p* = 0.276). However, when analyzing female patients separately, the AUC increased to 0.713 (*p* = 0.037, 95% CI: 0.539–0.887), indicating moderate predictive utility. The Hosmer–Lemeshow goodness‐of‐fit test confirmed good calibration of the model, with an *χ* ^2^ value of 5.012 (*p* = 0.756). The optimal threshold for predicting favorable intubation conditions was identified as an SPI value of 19, which demonstrated a sensitivity of 81.8% and a specificity of 46.9%. In contrast, SPI values at T2 did not effectively predict tracheal intubation conditions in male patients, with an AUC of 0.467 (*p* = 0.752), as summarized in Table 3.

**TABLE 3:** Predictive value of SPI on tracheal intubation conditions.

Group specificity	AUC (95% CI)	Cutoff	*p*	Sensitivity	Specificity
Overall population	0.579 (0.442–0.716)		0.276		
Female	0.713 (0.539–0.887)	19	0.037	81.8%	46.9%
Male	0.467 (0.272–0.662)		0.752		

Abbreviations: AUC, area under the curve; CI, confidence interval.

## 4. Discussion

This study assessed the predictive value of the SPI for assessing tracheal intubation conditions in the absence of NMBAs. Tracheal intubation is a significant nociceptive stimulus [21, 22], and the SPI is used to assess the balance between surgical pain stimuli and the level of analgesia provided during surgery [23, 24]. A key advantage of the SPI is its ability to provide an objective, real‐time measure of this balance, which may be particularly valuable in cases where NMBAs are not used to facilitate intubation [25, 26]. By investigating the relationship between SPI values immediately before intubation and the actual intubation conditions, this study determined whether SPI could serve as a reliable predictor of intubation quality, considering that reducing reliance on neuromuscular blockers could improve patient safety by minimizing associated adverse effects, such as postoperative myalgia, prolonged recovery, or intraoperative complications [27]. Furthermore, optimizing intubation conditions without neuromuscular blockers could enhance the efficiency of anesthesia and offer a more individualized approach to pain management and muscle relaxation during surgery.

This observational study involved 100 patients who underwent elective surgery under general anesthesia at The Affiliated Lihuili Hospital of Ningbo University, Ningbo, China, between November 2021 and June 2022. All participants successfully completed the study and met the inclusion and exclusion criteria outlined in the Methods section. The potential impact of patients’ physical status on the experimental outcomes was carefully considered, as factors such as age, weight, and overall health can influence responses to anesthesia and intubation. The cohort had a mean age of 48 years, with 47 males and 53 females, and an average weight of 64.4 kg. According to the ASA physical status classification system, 71% of patients were classified as ASA I, while 29% were classified as ASA II. The ASA classification is widely used to assess preoperative health status [28], predict perioperative risks, and guide anesthetic management [29]. ASA I refers to healthy patients with no systemic disease, while ASA II refers to patients with mild systemic disease that does not affect normal function [30]. Most participants were in good health with minimal comorbidities, consistent with the study’s inclusion criteria. The balanced gender distribution and the overall health status of the cohort suggest that physical factors did not significantly impact the experimental outcomes [31] and support the validity of the study’s results, ensuring reliable conclusions can be drawn.

To further ensure the reliability of our findings, the psychological status of the 100 participants was assessed using the HARS before surgery and classified anxiety levels as follows: 0–7 points indicating no anxiety, 7–17 points indicating mild to moderate anxiety, 18–24 points indicating moderate anxiety, and 25 or more points indicating severe anxiety [32]. The assessment was conducted in accordance with the methodology described by Sulastri et al. [18]. Our results indicated that most of the participants experienced either no anxiety or mild anxiety, suggesting that psychological factors were unlikely to have a significant impact on the study’s outcomes and that the psychological state of the participants was generally stable, thereby further reinforcing the validity and accuracy of the study’s findings.

Immediately before intubation, significant decreases in SBP, DBP, HR, and SPI were observed compared to baseline values recorded after the administration of Ringer’s solution and upon loss of consciousness, which suggests that an effective balance between nociceptive stimuli and analgesic effects was achieved, providing optimal conditions for intubation [33]. Notably, SPI reached its lowest value immediately before intubation, indicating that patients were sufficiently anesthetized to tolerate the procedure without the need for neuromuscular blockers [34]. Post‐intubation, physiological responses were found to vary. Both SBP and DBP significantly increased 3 minutes after intubation compared to pre‐intubation values, which reflects the typical physiological response to the intubation stimulus. However, HR and SPI remained stable, with no significant changes immediately after intubation. The stability of SPI post‐intubation suggests that the initial balance between nociceptive input and analgesia was maintained, reinforcing SPI’s utility in predicting and ensuring optimal intubation conditions [35] and supporting the hypothesis that SPI values immediately before intubation can reliably predict intubation conditions. The significant reduction in SPI immediately before intubation, followed by its stability post‐intubation despite the increase in blood pressure, supports the role of SPI in assessing the adequacy of anesthesia [36] and aligns with the primary objective of our study, which was to demonstrate that SPI can reliably predict optimal intubation conditions without the use of neuromuscular blockers.

Intubation conditions were evaluated using the criteria established by Erhan et al. [20], classifying them as excellent, good, or poor. All patients successfully underwent intubation, with 58 patients rated as “excellent”, 42 as “good”, and none as “poor”, which suggests that most patients experienced optimal or near‐optimal intubation conditions without the use of neuromuscular blockers [37]. A significant difference in SPI values was observed between the “excellent” and “good” groups. Specifically, SPI values were significantly higher in the “good” group compared to the “excellent” group immediately after intubation, 1 min post‐intubation, and 3 min post‐intubation, indicating that lower SPI values were associated with better intubation conditions [38]. The differences in SPI values between the two groups support SPI’s ability to predict intubation quality, whereby lower SPI values before intubation were associated with better intubation conditions, confirming our hypothesis.

Furthermore, respiratory parameters, including RR, EtCO_2_, and SpO_2_, were continuously monitored throughout the procedure to ensure patient safety and achieve optimal anesthesia management [39]. RR declined from T0 to T2 and was subsequently maintained at 12 bpm between T2 and T5. This pattern indicates reduced RR following anesthesia induction, with machine‐assisted ventilation stabilizing the rate at 12 bpm post‐intubation, suggesting both smooth intubation and an absence of stress responses. EtCO_2_ peaked at T3 but decreased with patient stabilization post‐intubation, confirming effective ventilation. SpO_2_ remained stable within predetermined safe thresholds after T1 due to prompt manual ventilation adjustments. These findings collectively support SPI’s utility as a predictive tool for assessing physiological responses during tracheal intubation without NMBAs [40].

The biochemical markers analyzed in this study provide additional insight into the physiological response to tracheal intubation performed without neuromuscular blockers. Specifically, cortisol levels were significantly elevated at T5 compared to T1, indicating a pronounced stress response associated with the intubation procedure. Cortisol, a primary stress hormone, typically rises in response to both physical and psychological stressors, such as those encountered during intubation [41]. The positive correlation between cortisol elevation and suboptimal intubation conditions suggests that SPI may indirectly reflect the stress response to intubation, further suggesting that SPI may serve as an effective predictor of stress responses during intubation [35]. In contrast, CRP levels did not show significant changes between T1 and T5, suggesting that the acute‐phase inflammatory response may not be as immediately impacted by the stress of intubation. CRP is generally associated with longer‐term or sustained inflammation, and thus, may not be as responsive to the short‐term stress induced by procedures like intubation [42]. This distinction highlights the utility of SPI in predicting immediate stress responses (as indicated by cortisol), while it may be less sensitive to the inflammatory changes reflected by CRP.

We conducted an ROC curve analysis to assess how well the SPI could predict tracheal intubation conditions, and used AUC as a measure of the SPI’s predictive ability [33]. For the overall group, the SPI at the T2 time point had an AUC of 0.579 (*p* = 0.276), which indicates that the SPI at this time point was not a reliable predictor for intubation conditions. However, when we analyzed the results by gender, we observed a significant difference. In female patients, the AUC was 0.713 (*p* = 0.037), suggesting that an SPI value below 19 at T2 was associated with better intubation conditions. In contrast, the SPI did not show predictive value in male patients, as their AUC was 0.467 (*p* = 0.752), meaning it was not able to predict intubation conditions in this group. These findings suggest that SPI has a gender‐specific predictive value, being more reliable for predicting intubation conditions in females than in males. It should be noted that the sex‐stratified ROC analysis was exploratory and post hoc. While the results showed a moderate predictive value of SPI in female patients, this finding requires confirmation in prespecified, hypothesis‐driven studies. The exploratory nature of this analysis limits the strength of the conclusion, and caution should be exercised when generalizing the gender‐specific findings.

The optimal SPI cutoff value of 19 for predicting favorable intubation conditions in female patients showed a sensitivity of 81.8% but a relatively low specificity of 46.9%. This low specificity indicates that a considerable proportion of patients with SPI values above 19 may still have favorable intubation conditions, limiting the clinical utility of this cutoff as a standalone predictor. The low specificity may be attributed to individual differences in physiological responses to anesthesia, as well as other unmeasured factors (e.g., genetic variations, preoperative medication use) that can influence intubation conditions. In clinical practice, the SPI cutoff should be used in conjunction with other clinical assessments (e.g., BIS values, clinical judgment of anesthesiologists) to improve the accuracy of predicting intubation conditions. Future studies could explore combining SPI with other physiological parameters to develop a more comprehensive predictive model with higher specificity.

Several factors may explain the observed gender differences in the effectiveness of SPI as a predictor of tracheal intubation conditions when neuromuscular blockers are not used. Physiological and hormonal disparities between males and females can influence how patients respond to anesthesia, pain, and other physiological stimuli [43]. For instance, gender differences in brain structure and function may affect how the nervous system processes pharmacological agents, which could, in turn, impact the accuracy and reliability of SPI as a predictor of intubation conditions. Moreover, females may exhibit increased sensitivity to certain stimuli, such as pain, which could influence their perception of discomfort and contribute to greater variability in physiological parameters, including HR and blood pressure. This increased sensitivity may enhance the predictive value of SPI in females, while reducing its effectiveness in males [44, 45]. In addition, these gender‐specific physiological responses and sensitivities likely underlie the differential predictive value of SPI observed between male and female patients during tracheal intubation assessments [46, 47]. But these mechanisms are speculative and should be considered hypothesis‐generating. These hypotheses require further investigation using targeted studies (e.g., neuroimaging studies, hormonal analysis) to confirm and elaborate. The current study provides preliminary evidence for gender‐specific differences but cannot establish causal relationships between physiological mechanisms and SPI predictive performance.

While these findings demonstrate the reliability and effectiveness of the SPI as a tool for assessing tracheal intubation conditions in female patients without NMBAs, several important limitations must be acknowledged. First, the study was conducted at a single institution with a relatively homogeneous patient population (ASA I‐II, no anticipated intubation difficulties), and no patients were rated as having “poor” intubation conditions—a factor that limits outcome discrimination and generalizability to higher‐risk populations. Second, the observational design is inherently susceptible to uncontrolled confounding, which may affect interpretation of the results. Additionally, the limited sample size and lack of multifactorial analysis to clarify gender‐specific mechanisms in SPI’s predictive performance further constrain the robustness of the conclusions.

Future research should prioritize multicenter validation with larger, more diverse patient cohorts, including those with anticipated difficult intubation, to enhance generalizability and assess SPI across all intubation condition grades. Further investigation into the mechanisms underlying gender differences using complementary biomarkers or physiological parameters is also warranted. Finally, randomized controlled trials are needed to strengthen causal inference and confirm the predictive utility of SPI in varied clinical settings.

## 5. Conclusions

**FIGURE 8:**
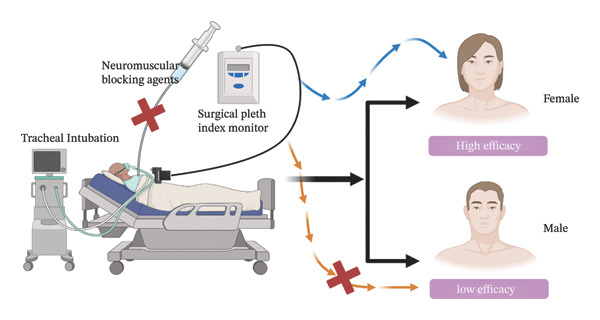
Predictive value of the SPI for tracheal intubation conditions in the absence of neuromuscular blocking agents.

In conclusion, this study demonstrates the potential utility of SPI as a simple and noninvasive tool for evaluating tracheal intubation conditions in female patients without the use of neuromuscular blockers, as it was effective in improving intubation success and patient satisfaction within this subgroup. However, its predictive value is moderate, with a relatively low specificity, and it should be used in conjunction with other clinical assessments in practice. The predictive value of SPI for male patients remains inconclusive, warranting the need for further research to explore the underlying reasons for these gender‐based differences and to refine SPI’s clinical applicability (Figure 8). Future studies could continue to investigate factors contributing to these gender disparities and seek to optimize SPI’s role in clinical practice. Caution should be exercised when generalizing the results due to the observational nature of the study, lack of external validation, and exclusion of patients with poor intubation conditions.

## Author Contributions

Jiale Chen, Zhihao Pan, and Jinwei Zheng contributed to the conceptualization. Jiale Chen performed the analysis and drafted the first version of the manuscript. All authors contributed to data acquisition. Zhihao Pan participated in data acquisition and analysis and critically revised the manuscript for important intellectual content. Jinwei Zheng supervised the study, provided administrative support, and ensured the accuracy and integrity of the work.

## Funding

The authors have nothing to report.

## Disclosure

All authors commented on previous versions of the manuscript and approved the final version for publication.

## Ethics Statement

The study protocol was approved by the Research Ethics Committee of the Affiliated Lihuili Hospital of Ningbo University (Ethical Application Reference: KY2021SL179‐01, Ningbo, China) on 29 October 2021. All methods were conducted in accordance with relevant ethical guidelines, regulations, and CONSORT recommendations. Prior to participation, written informed consent was obtained from all patients and/or their legal guardians.

## Conflicts of Interest

The authors declare no conflicts of interest.

## Data Availability

The datasets used and/or analyzed during the current study are available from the corresponding author upon reasonable request.
